# Future applications of host direct therapies for infectious disease treatment

**DOI:** 10.3389/fimmu.2024.1436557

**Published:** 2024-10-01

**Authors:** Ruth E. Thom, R V. D’Elia

**Affiliations:** ^1^ Chemical, Biological and Radiological Division, Defence Science and Technology Laboratory, Porton Down, Salisbury, United Kingdom; ^2^ Strathclyde Institute of Pharmacy & Biomedical Sciences, University of Strathclyde, Glasgow, United Kingdom

**Keywords:** host-directed, therapeutic, STING, pyroptosis, itaconate, infection

## Abstract

New and emerging pathogens, such as SARS-CoV2 have highlighted the requirement for threat agnostic therapies. Some antibiotics or antivirals can demonstrate broad-spectrum activity against pathogens in the same family or genus but efficacy can quickly reduce due to their specific mechanism of action and for the ability of the disease causing agent to evolve. This has led to the generation of antimicrobial resistant strains, making infectious diseases more difficult to treat. Alternative approaches therefore need to be considered, which include exploring the utility of Host-Directed Therapies (HDTs). This is a growing area with huge potential but difficulties arise due to the complexity of disease profiles. For example, a HDT given early during infection may not be appropriate or as effective when the disease has become chronic or when a patient is in intensive care. With the growing understanding of immune function, a new generation of HDT for the treatment of disease could allow targeting specific pathways to augment or diminish the host response, dependent upon disease profile, and allow for bespoke therapeutic management plans. This review highlights promising and approved HDTs that can manipulate the immune system throughout the spectrum of disease, in particular to viral and bacterial pathogens, and demonstrates how the advantages of HDT will soon outweigh the potential side effects.

## 1 Introduction to host-directed therapies

Since the beginning of the 20^th^ century and the advent of antibiotics the premise to treat infectious disease is the use of antimicrobial agents that directly target the pathogen. To our detriment, now in the 21^st^ century we are still heavily reliant on this approach and we are continually facing new strains of bacteria and viruses that are resistant to our available armament. Furthermore, lessons learnt from the coronavirus disease 2019 (COVID-19) global pandemic mean we need to become better equipped for the emergence of new infectious disease.

Research and development into alternative solutions for the treatment of infectious disease has accelerated and one such approach is to identify drugs that modulate the host pathways in a growing area of research known as Host-Directed Therapies (HDTs). HDTs are showing success in the field of cancer with a number of licenced products (1, 2). For infectious disease, momentum is building to develop HDTs and it is becoming a promising area of drug discovery. HDTs are much less prone to the generation of drug-resistant pathogen strains because the therapeutic strategy is to target evolutionary conserved host factors. The pathogen would require considerable evolutionary changes to overcome these targeted host pathways (3). HDT could also offer a broad-spectrum of therapy and would be beneficial where rapid treatment is required such as during epidemics and pandemics as well for the preparedness of new pathogens.

The advantages of HDT do need to be caveated for the potential of toxicity. Indeed targeting host-specific pathways could have devastating effects on the host as seen in the first phase 1 clinical trial of an agonistic anti-CD28 monoclonal antibody, which led to an incapacitating cytokine storm in the volunteers (4). Furthermore, the therapeutic window for HDT is critical in the treatment strategy. For example, it was reported that the early treatment of COVID-19 patients with exogenous IFNα was beneficial (5, 6), but was detrimental when administered later in disease (7, 8). To overcome this careful monitoring of the host and understanding of the time course of infection is critical. This can be achieved with the use of diagnostic biomarkers which can differentiate between bacterial and viral infection (9) as well as pre-symptomatic diagnosis of cytokine storms including biomarkers of sepsis (10).

HDT encompass a continually growing arsenal of agents, which includes repurposed drugs, small molecules, synthetic nucleic acids, biologics, cytokines, cellular therapy, recombinant proteins and micronutrients (11). Here we describe a range of HDT strategies, which is not exhaustive, but provides a representation of the research and development in this field focussing on infectious disease caused by bacterial and viral pathogens. The application of HDT for fungal and parasitic infections are reviewed in detail elsewhere (12–15). An area that will not be discussed will be therapeutic and prophylactic vaccination and the overview will focus on alternative methods to modify the host response. We have compartmentalised the course of disease into specific phases to describe the potential beneficial uses for HDT: (i) Early phase, referring to pathogen entry and establishment of infection. (ii) Middle phase, including disease progression leading to either convalescence or acute infection. (iii) Late phase, which describes persistency and latency. However, some therapies or targets may have applicability across more than one phase of infectious disease. For instance, it may also be advantageous to boost the immune response when the disease has reached latency and not just early in infection; such examples will be discussed. Further, we conclude that with increased depth of knowledge of immune function across the time course of infection, the same HDT pathway could be manipulated to either agonise or antagonise host defence responses supporting a protective outcome over the spectrum of disease.

## 2 Early intervention using HDT to treat infectious disease

**Figure 1:**
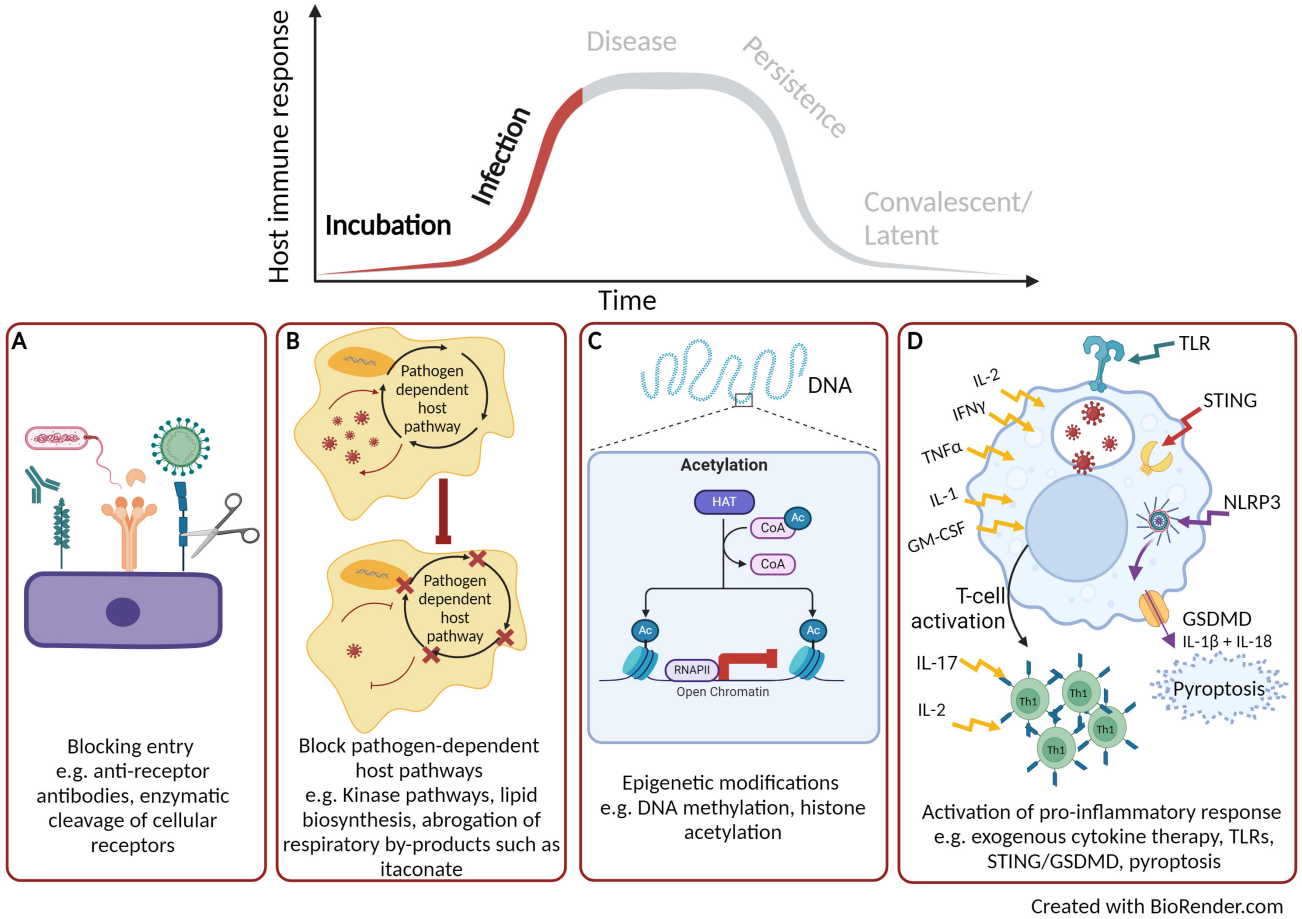
Early targets for host directed therapies. **(A)** Blocking pathogen entry. **(B)** Blocking pathogen-dependent host pathways. **(C)** Host or pathogen epigenetic modification. **(D)** Activation of pro-inflammatory cytokine response. Created with BioRender.com.

The earliest point to target the host upon pathogen infection is to block or inhibit cellular entry thus rendering the host cell non-permissive (**Figure 1A**). With advancements in the understanding of host-pathogen interactions, novel HDT strategies targeting pathogen entry are currently being pursued. The most progress has been achieved with the treatment of Human immunodeficiency virus (HIV)-1 by targeting CC-chemokine receptor 5 (CCR5). CCR5 is a cofactor for the entry of the virus and antagonists of CCR5 inhibit its function and can block viral entry (16). Maraviroc was the first CCR5 antagonist to be licenced in 2007 and has now become part of the therapeutic schedule for HIV positive patients (17). Additionally entry inhibitors for hepatitis B and D viruses are also now licenced, such as myrcludex B (18); illustrating the promise that entry inhibitors are successful HDT targets (19, 20). The identification of other early entry molecules for harmful viruses such Ebola virus (21) and Lassa virus (22) is a starting point for potential HDTs. In the case for Ebola, a number of small molecules have been identified that can affect various stages of Ebola virus uptake from cell attachment, internalisation by macropinocytosis and fusion of the viral envelope (23). Madrid et al. (24) demonstrated that the chloroquine (an approved antimalarial treatment) can inhibit the trafficking of the Ebola virus through the endosomal pathway and prevents viral fusion thus aborting infection. Using a murine model of Ebola infection, treatment with chloroquine led to 80-90% survival (24). Inhibiting pathogen entry pathway could be beneficial as a pre-exposure therapy for instance during an epidemic or pandemic. They could also be utilized to negate subsequent rounds of pathogen entry and replication thus alleviating the infectious cycle.

Another attractive approach for HDT development is to target cellular pathways that the pathogen is dependent upon for replication and infection but are dispensable to the host (**Figure 1B**). Targeting host pathogen-dependent pathways, instead of individual factors, is a more promising HDT approach for bacterial infections owning to its higher autonomy compared to viruses. The majority of research has focused on kinases and lipid biosynthesis. There are over 500 kinases identified by the Human Genome Project, which are involved in a range of physiological processes and cellular homeostasis (25). Kinases are also associated with all stages of viral replication, however, a number of cellular kinases have been identified to be non-essential for the host but are required for viral infection (26). Such kinases represent potentially valuable drug targets. Kinase inhibitors are small chemical molecules and the screening of kinase inhibitor libraries has identified some promising HDT candidates that are required for pathogen replication but are non-essential to the host. Inhibitors to two receptor tyrosine kinases have been discovered that block the replication of a range of DNA (herpes simplex virus) and RNA (influenza A virus, Sendai virus, mouse hepatitis virus and rhesus rotavirus) viruses (27). A whole range of kinase inhibitors have been licenced for the treatment of cancer therapy (28) and these compounds are now been examined for use as HDT. For example, dasatinib, a potent inhibitor of the SCR kinases, is used in the treatment of chronic myeloid leukaemia. However, repurposing of dasatinib has also shown beneficial effects in preventing dengue virus replication by inhibition of viral RNA replication and particle secretion (29). During the COVID-19 pandemic, a number of licenced kinase inhibitors were identified for both inhibition of viral life cycle [e.g. tyrosine kinase inhibitor, imatinib (30)] as well as those that could reduce host immuno-pathology [e.g. Janus kinase inhibitor, baricitinib (31)]; further demonstrating the potential broad-range activity of kinase inhibitors as HDTs (32). Fatty acids are required for pathogens to replicate and they can gain these host factors by reprogramming cellular metabolism, including lipid synthesis (33). Blocking lipid synthesis with chemical inhibitors has been shown to decrease the production of flaviviruses (34). Chu et al. (35) screened 22 fatty acid inhibitors to identify compounds that could inhibit replication of SARS-CoV2 and demonstrated that half of the compounds could significantly reduce replication *in vitro*. The most prominent was orlistat, which is a licenced anti-obesity drug that reduces the absorption of dietary fat through the inhibition of lipases. Chu et al. (35), demonstrated in a SARS-CoV2 murine model that following treatment with orlistat there was reduced viral loads within the lungs, reduced lung pathology and increased survival (35). *Mycobacterium tuberculosis* resides in macrophages and requires fatty acids derived from lipid bodies as an essential source of energy. The lipid sensing nuclear receptor, peroxisome proliferator-activated gamma (PPARγ), can be activated by mycobacteria to form lipid bodies (36). Pre-treatment of macrophages with a PPARγ antagonist followed by mycobacterial infection leads to a decrease in lipid body formation as well effective mycobactericidal activity (37).

Bacterial pathogens have also been reported to utilise by-products of the host cellular respiration cycle to support growth. For instance, itaconate, a small metabolic molecule that is a by-product of the tricarboxylic acid (TCA) cycle, is known to have direct links to immune function (38) and has a range of anti-inflammatory and anti-oxidant functions (39, 40). Despite the antimicrobial properties of itaconate, intracellular bacteria have developed strategies to benefit from endogenous itaconate (41). For example, *Klebsiella pneumoniae*, can induce metabolic oxidative stress responses through lipopolysaccharide binding to toll-like receptor (TLR) 4 leading to the accumulation of itaconate. This bacterial defence mechanism has been shown to promote an anti-inflammatory response and induce a disease-tolerant immune response (42). Furthermore, some bacterial pathogens such as *Psuedomonas aeruginosa* and *Staphylococcus aureus* utilise itaconate as a carbon source to establish a persistent infection and support the development of biofilms (43, 44). The production of itaconate can be controlled by the immune response gene 1 (IRG1) and the utilization of itaconate by pathogens to tolerate the host response and to support growth is achieved through the activation of the IRG1 pathway (39). Further research is required to unravel the host-pathogen link with IRG1-itaconate, but there is potential for HDT to target this pathway and abrogate the utilization of itaconate by pathogens (**Figure 1B**).

As well as targeting pathogen-dependent host factors, directing HDTs towards DNA-modifying enzymes is an alternative approach under development (**Figure 1C**). Phenotypic modification of genomic DNA caused by DNA methylation and histone acetylation leads to altered structures and stability of the DNA which can regulate gene expression and cell division (45). These DNA-modifying enzymes have been used in the successful treatment of cancers (46). For example, vorinostat was the first approved histone deacetylase inhibitor to be used as a therapy to treat cutaneous T-cell lymphoma (47). Human macrophages infected with *Salmonella enterica* alongside treatment with an inhibitor of histone deacetylase have shown to promote intracellular bacterial clearance through the induction of mitochondrial reactive oxygen species (ROS) (48). Additional studies have demonstrated the inhibition of histone deacetylase can subvert the cytotoxic effects of bacterial toxins, such as those produced by *Bacillus anthracis*. Macrophages treated with a histone deacetylase inhibitor following exposure to *B. anthracis* lethal toxin showed a marked increase in pro-inflammatory cytokines signalling pathways such as IL-1β as well as pyroptosis, a pro-inflammatory programmed cell death pathway (49). Conversely, pathogens can also target these host enzymes to modify the host genome and become permissive to infection. For example *B. anthracis* and *M. tuberculosis* have both been reported to modulate histone phosphorylation of down-stream inflammatory pathways resulting in alterations in macrophage and epithelial cell activation (50, 51); thus efforts to develop inhibitors to these pathogen DNA modification pathways are also on going (52, 53).

The HDT approaches described above have focussed on enhancing underlying antimicrobial cellular pathways that aim to control and clear infection. An alternative strategy is to target the early host immune response (**Figure 1D**). Since the second half of the 20^th^ Century, there have been numerous examples of the use of exogenous cytokine therapy for the treatment of viral infections such as influenza (54), hepatitis C (55) and HIV-1 (56) right up until the present day with the treatment of COVID-19 (57). A number of successful recombinant interferons have been licenced, such as IFN-alpha2b (licenced as Intron A) for the treatment of hepatitis B and C, as well as human papillomavirus (58) and early infection to SARS-CoV2 (57). The use of exogenous cytokines have been well documented for the treatment of tuberculosis, for example cytokines TNFα, IFNγ and IL-1α are known to stimulate antimicrobial properties of mycobacterial infected macrophages (59). The delivery of IFNγ via the aerosol route, in combination with standard therapy, demonstrated promising results for patients with multi-drug resistant pulmonary *M. tuberculosis* infection. The study reported that the combinational therapy led to enhanced mycobacterial killing, reduced lung lesions and improved clinical outcome (60). Exogenous cytokine therapy can have diverse effects on the host immune response including the activation and recruitment of immune cells as well as down-stream signalling to amplify the antimicrobial immune response. However, there can be severe side effects to cytokine-based therapy and timing is critical to when they should be administered. Cytokine therapy remains a key research interest in cancer therapy, with an IFNα (Peginterferon-α 2b) and IL-2 (Aldesleukin) therapies approved for specific cancers (61). Recent advancements in cytokine-based therapeutics, such as improving half-life, targeted delivery and reduced toxicity, still make them an appealing HDT. New technology and improved understanding of pharmacodynamics/pharmacokinetics has led to bio-engineered cytokines that can be directed to the site of immunopathology in a timely manner (62). Furthermore, advances in the individual treatment of patients can lead to bespoke individual management plans (63). Alternatively, endogenous cytokines can be induced by the activation of TLRs (64), for example, imiquimod is a TLR7 agonist that is used to treat human papillomavirus. When it is applied topically to warts, imiquimod activates IFNα, IL-1, IL-6, and TNFα leading to the reduction of viral load (65).

Early innate immune responses rely on the detection of conserved structural features of the pathogen, known as pathogen-associated molecular patterns (PAMPs) by binding to host pattern-recognition receptors (PRRs), present on the cell surface or within the cells. Over the last decade DNA and RNA sensing PPRs have been described which are typically activated through viral infection leading to a potent antiviral host immune response. Such PPRs include; TLRs, RIG-1 like receptors (RLRs), NOD-like receptors (NLRs) and cyclic GMP-AMP synthase (cGAS) protein families, all of which have been extensively reviewed (66, 67). More recently it has been identified that these nucleic acid sensing pathways could be a potential target for HDTs (68). Indeed, cGAS which senses both self and foreign double-stranded DNA activates the cGAS-stimulator of interferon genes (STING) signalling pathway resulting in the expression of type 1 IFNs (69, 70). The cGAS-STING signalling pathway is critical in the activation of the innate immune response, but in addition, an increasing number of immune roles have been described (71). Conversely, RNA viruses (including Dengue virus, Influenza A Virus, Zika virus and SARS-CoV2) have been reported to antagonise cGAS-STING and block DNA-dependent IFN-1 activation (72). Thus, during infection with RNA viruses, the release of host genomic or mitochondrial DNA within the cytoplasm would not be detected and cGAS-STING-induced anti-viral immune responses will be inhibited. STING agonists have been identified that induce cGAS-STING signalling prior to and during early infection of RNA viruses (**Figure 1D**). Humpries et al. (73) administered the STING agonist, diABZI-4 intranasally to a SARS-CoV2 murine model and demonstrated transient activation of STING. They reported a pro-inflammatory response, with cytokine production, lymphocyte activation and inhibition of viral replication (73).

Exploitation of pyroptosis, a rapid and lytic pro-inflammatory programmed cell death pathway, has been shown to be another effective early HDT for infectious disease (**Figure 1D**). Upon activation of either PAMPs (e.g. bacterial derived molecules and viral nucleic acid) or damage-associated molecular pattern (DAMPs, host molecular makers of disease e.g. ATP, IL-1α, DNA) leads to a cascade of events resulting in the assembly of cytosolic pro-inflammatory complexes such as the NOD-, LRR- and pyrin domain-containing protein 3 (NLRP3) inflammasome (74). NLRP3 activates the inflammatory cytokines IL-1β and IL-18 as well as the pore-forming protein, gasdermin D (GSDMD). Initially, the GSDMD pore allows the release of these cytokines from macrophages and dendritic cells but ultimately leads to pyroptosis through osmotic cell lysis and disruption of the plasma membrane (75). GSDMD has an essential role in innate immunity; inducing a pro-inflammatory response, promoting effective pathogen clearance and preventing replication (76). Indeed, the induction of pyroptosis by GSDMD has been shown to protect a melioidosis murine model following infection with the intracellular bacteria, *Burkholderia thaliandensis* (77). Furthermore, antibody-opsonised SARS-CoV2 infection of human blood monocytes and macrophages activates the NLRP3 inflammasome, inducing pyroptosis, as demonstrated by increased levels of GSDMD and IL-18. Pyroptosis occurs rapidly preventing the replication and assembly of infectious viral progeny thus rendering myeloid cells a dead end for infection (78). In some cases, a pathogen can hijack the process of pyroptosis, such as the case for intracellular *M. tuberculosis* infection, where the cellular membrane is disrupted and impairs GSDMD-mediated pyroptosis (79). Exploiting this rapid innate immune-regulated form of cell death through activation of NLRP3 signalling via DAMPs or PAMPs could be an effective early HDT to protect from infectious disease. Pre-clinical cancer therapies targeting pyroptosis is currently leading the way in this approach with several different therapy strategies (80). Alternative licenced drugs such as metformin (for diabetic treatment) and ivermectin (an anti-parasitic agent) have been demonstrated to induce pyroptosis and exert anti-tumour activity *in vitro* and *in vivo* (81, 82). These studies are examples of how licenced drugs have the potential to be repurposed for other diseases.

## 3 The use of HDT to induce immune homeostasis and minimise immunopathology during disease progression

As disease-causing pathogens establish infection and evade the early innate host immune response, the adaptive immune response begins to develop, initiating an antigen-specific cellular and/or humoral infiltration. During disease progression, the innate and adaptive immune responses are not mutually exclusive but are complementary in the resolution of disease. Strategies to enhance the adaptive immune response can prevent the establishment of latent or persistent infection and support the immune cells in eliminating infectious pathogens. Such strategies include vaccination, cytokine therapy, adoptive cell transfer and immune checkpoint blockade (the later discussed below) (83). If these two arms of the immune response are not aligned then the host response can become dysregulated resulting in tissue damage caused by immunopathology, acute disease status and morbidity. In this section, we discuss HDTs that can rebalance the host immune response thus reducing disease severity and eliminate infectious pathogens.

**Figure 2:**
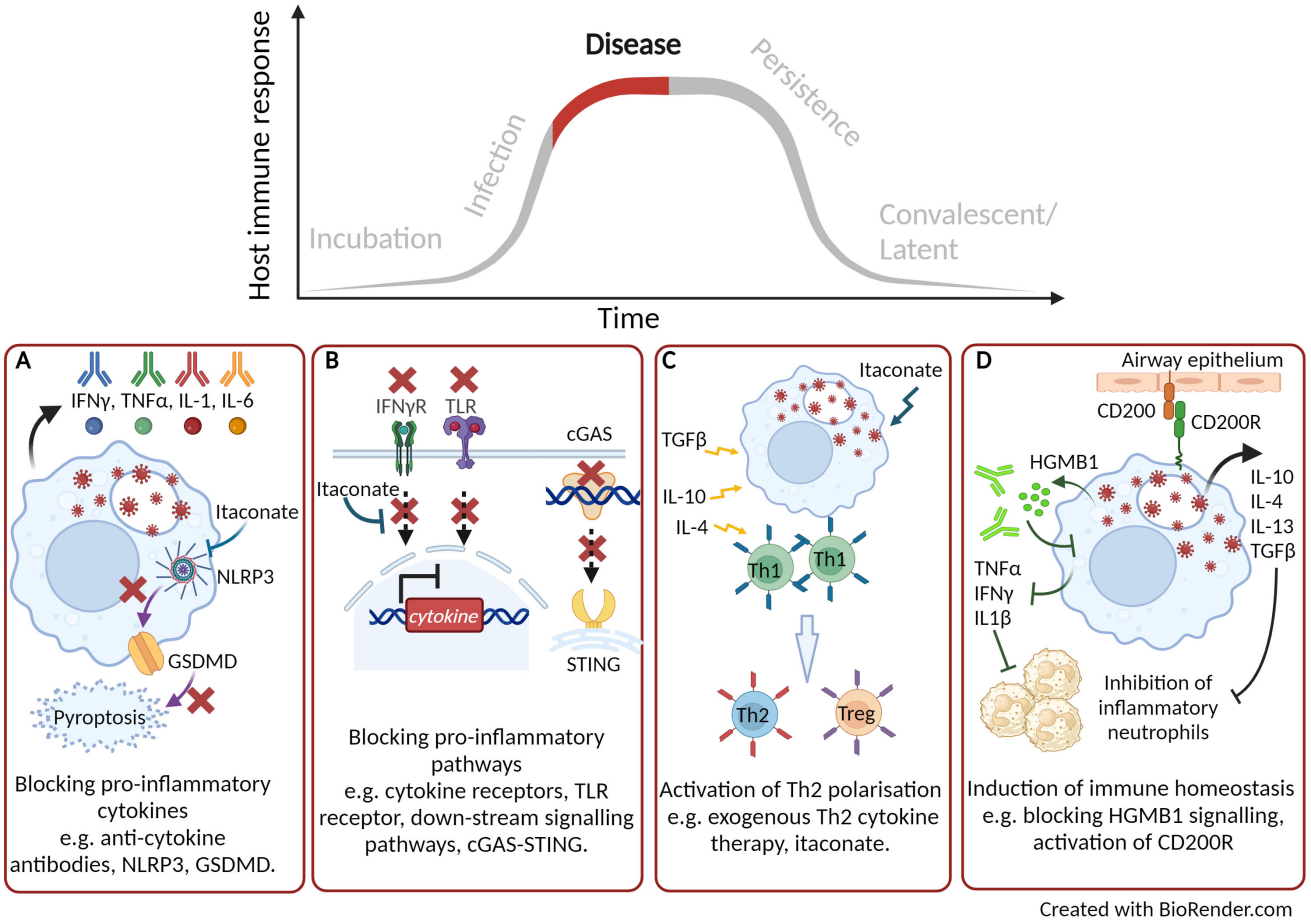
Targeting host directed therapies to treat and reduce disease progression. **(A)** Blocking pro-inflammatory cytokines. **(B)** Blocking down-stream signalling pathways. **(C)** Activation of Th2 immune response. **(D)** Induction of immune homeostasis. Created with BioRender.com.

It is well reported that during some acute and severe infections, a cytokine storm can be activated which correlates with increased disease severity and mortality (84). Over 150 cytokines have been reported to be involved in a cytokine storm but primarily the key cytokines are TNFα, IL-6 and IFN’s (85). In some cases, treatment with a monoclonal antibody directed towards one of these cytokines can have beneficial therapeutic effect (**Figure 2A**). During the COVID-19 pandemic monoclonal antibodies targeting IL-6, IL-1β, IL-23 and GM-CSF, or their receptors, went through clinical trials and demonstrated varying levels of therapeutic efficacy by reducing morbidity and mortality (86). A number of clinical trials have demonstrated the use of tocilizumab, an anti-IL-6 compound, as a COVID-19 therapeutic (87). The largest of these clinical trials, (RECOVERY), reported the most compelling evidence of the benefit to treat patients with acute infection with tocilizumab, leading to improved clinical outcome and an increase likelihood to be discharged from hospital within 28 days (88). To block the down-stream signalling pathways that activate pro-inflammatory cytokines and cytokine storms maybe a more effective HDT approach (**Figure 2B**). The transcription factor nuclear factor-kappa beta (NF-κβ) is critical in the regulation and downstream signalling pathways of cytokines involved in both the innate and adaptive immune response. Targeting this transcription factor has been shown to have therapeutic advantages in a mouse model of influenza strain H5N1 leading to a drastic reduction in NF-κβ regulated cytokines (89). The inhibition of NF-κβ signalling has also been an effective target to reduce the inflammatory response during critical stages of SARS-CoV-2 infection (90). As part of the COVID-19 RECOVERY trial, the therapeutic benefits of the anti-inflammatory corticosteroid dexamethasone were assessed using either a high or a low dose to treat patients on respiratory support (91). Dexamethasone is used for a broad range of inflammatory conditions and is known to supress NF-κβ (92). The COVID-19 patients on respiratory support that received the lower dose of dexamethasone demonstrated significant protection with 20-30% reduced mortality (91). However, the study was stopped due to an increase in mortality seen in patients receiving the high-dose therapy. It was hypothesised that due to an excessive dampening of the of the immune response, there was an increase opportunity for secondary infections (91). Inflammatory responses have also been demonstrated to be dampened by treatment of the DNAzyme Dz13 which is known to cleave the transcription factor c-Jun (93). c-Jun is activated during the early stages of influenza A and is involved in viral replication as well as induction of the inflammatory response. Administration of Dz13 *in vivo* following influenza A infection resulted in significantly improved survival, as well as decreased viral titres and reduced production of pro-inflammatory cytokines in lung tissues (94). In the field of cancer therapy, a number of approved proteasome inhibitors (such as bortezomib, carfilzomib and ixazomib) are known to be strong suppressors of down-stream signalling pathways, such as NF-κβ (95). It is plausible that such therapies could be used in down-regulating acute cytokine storms induced by bacterial or viral infections. An alternative to blocking pro-inflammatory cytokine responses is to activate the Th2 immune response through exogenous Th2 cytokine therapy (**Figure 2C**), leading to immune homeostasis, protective immunity and tissue repair (96, 97). IL-10 therapy has had success for the treatment of inflammatory conditions, such as rheumatoid arthritis, psoriasis and inflammatory bowl disease (98). The most advanced IL-10 therapy has been the treatment of cancer patients with a PEGylated recombinant human IL-10 (PEG-rHuIL-10), which has been shown to suppress tumour-associated immunity, improve clinical outcome (99). Indeed IL-10 or agonists of the down-stream signalling pathways have been proposed as a therapeutic for acute lung infection with *Streptococcus pneumonia* (100), chronic mycobacterial infection (101) as well for COVID-19 therapy (102).

As described earlier, nucleic acid sensing pathways are critical in the activation of anti-viral innate immune response. However, these pathways can become dysregulated and depending on the intensity of the signal, a protective pathway could lead to a pathological outcome. Using a murine SARS-CoV2 infection model, Domizio et al. (70) demonstrated that the RNA virus promoted mitochondrial damage leading to mitochondrial DNA leakage. The presence of mitochondrial DNA within the cytosol of infected cells activated the cGAS-STING signalling pathway leading to inflammation and extensive lung pathology (70). They further demonstrated that treatment with the STING inhibitor, H151, in their murine model showed a decrease in lung inflammation at late time points and a reduction of viral loads. A number of high-throughput screening studies have identified antagonists of the cGAS-STING pathway which have been demonstrated to either inhibit cGAS (103) or STING though competitively binding at the substrate binding sites (104) or induce conformation change (105) (**Figure 2B**).

Pyroptosis, although a critical early innate host response that can prevent infection and replication of both bacteria and virus, can become a double-edged sword. Recent studies have revealed examples where chronic activation can have a detrimental role resulting in immunopathogenesis. In a murine model of severe influenza A infection, mice typically succumb to fatal pulmonary disease due to a hyper-inflammatory response and tissue damage (106). Using a *gsdmd ^-/-^* modified mouse model of severe influenza A infection, Rosli et al. (106), demonstrated a significantly improved outcome with increased survival, reduced viral burden and reduced tissue pathology compared to infection in wild type mice (106). Additionally, pyroptosis was shown to be a major cause of inflammatory sequelae in patients with critical COVID-19 symptoms, resulting in severe lung damage and multi-organ failure (78). HDTs are emerging which can target the NLRP3 inflammasome pathway (107). Pre-clinical studies using compounds that can inhibit either GSDMD and NLRP3 have been successful in the treatment of a range of immunopathological disease models, (108). The most widely researched NLRP3 inhibitor is a small molecule, MCC950, known to bind and lock the inflammasome in an inactive conformation (109). Using a murine infection model of influenza A, Tate et al. (110) demonstrated the timely importance of administering the NLRP3 inhibitor. When MCC950 was administered early after influenza A challenge, mice succumbed to fatal infection. However, when the inhibitor was used to treat mice later in infection, there was reduced inflammation within the lungs and prolonged survival (110). Targeting the pyroptotic cell death pathway such that GSDMD pores are reduced or inhibited, could be a potential new HDT to protect against disease caused by infectious pathogens (**Figure 2A**).

It is now becoming clear that cellular metabolic process, essential for biological function, can directly effect the outcome to infectious disease and inflammation (38). As described earlier the TCA by-product, itaconate, is known to have immuno-modulatory properties. In recent studies, itaconate has been shown to reduce inflammation by modification of pro-inflammatory inflammasomes, such as the NLRP3 inflammasome. Itaconate can modify the NLRP3 complex and ameliorate NLRP3 induced cascade of pro-inflammatory cytokines IL-1β and IL-18 (111) (**Figure 2A**). Itaconate has also been described to modulate immune responses though the activation or suppression of a range of transcriptions factors to limit pro-inflammatory cytokines (**Figure 2B**), induce antioxidant responses and regulate macrophage polarization (**Figure 2C**). For instance, the induction of the activating transcription factor (AFT3) through itaconate is reported to inhibit the production of pro-inflammatory cytokines (112). Furthermore, the nuclear factor erythroid 2-related factor 2 (NRF2) induces antioxidant and anti-inflammatory responses. The use of the itaconate derivative, 4-octyl itaconate, was shown to induce NRF2 and promoted a successful wound healing phenotype leading to a topical treatment for chronic wounds (113). Itaconate role in the regulation of macrophage polarization was also demonstrated through the suppression of Janus kinase 1 (JAK1) signalling (112, 114). Owning to the broad range of immunological function of itaconate, using a chemically synthesised derivative of the metabolite has demonstrated huge potential as a HDT therapy across both viral (Herpes Simplex Virus-1 and-2, Vaccinia virus, Zika virus and SARS-CoV2 (115)) and bacterial (*M. tuberculosis* (116)*, Francisella tularensis* (117)*, Brucella abortus* (118) and *Coxellia burnettii* (119) infections. Furthermore, there have been no known reports of pathogen utilization of these synthetic itaconate compounds unlike their endogenous counterparts (41).

In our laboratory, we are interested in immunomodulatory drugs that target the host and we have reported promising immunomodulatory data when reducing high mobility group B protein 1 (HMGB1). HMGB1 is a DAMP molecule and induces signalling of a pro-inflammatory cytokine response. It is released from damaged or infected cells and has been correlated to poor prognosis in human melioidosis patients (120). Using our *Burkholderia pseudomallei* mouse model, we have demonstrated that blocking HMGB1 signalling with a monoclonal antibody led to reduced bacterial burden in organ tissues which correlated to a reduction in pro-inflammatory cytokines (121). Similar findings were also reported in our *F. tularensis* mouse model (122) highlighting the potential of broad-range spectrum use of these immunomodulatory compounds.

Our more recent research investigating the immunomodulator CD200-Fc has also demonstrated effective treatment in mouse models of *F. tularensis* LVS (123) as well as in our murine aerosol models of CDC category A threat agents, such as *B. pseudomallei* (124). We hypothesised that CD200-Fc binds to its receptor and activates immune homeostasis through Th1 and Th2 cytokine profiles as well as inducing antimicrobial activity through the induction of ROS (**Figure 2D**). This work is further supported by data published demonstrating the importance of CD200 receptor in the lung macrophage following severe influenza infection by reducing lung inflammation and inducing immune homeostasis (125).

As discussed earlier, an overactive immune response can contribute to disease lethality. Even if the host is able to survive, it is likely that damage to cells and tissues has occurred leading to short or long-term immunopathology. Aiding the body to recover from tissue damage can significantly reduce morbidity and decrease the risk of secondary infections. Resolvins are a class of lipid metabolites that have been extensively studied which promote the resolution of chronic infection and used to treat a range of chronic inflammatory diseases, as previously reviewed (126, 127). The use of resolvins alongside the other HDT strategies discussed above could have a double benefit by reduce disease progression as well as protecting the host from immunopathology.

## 4 The use of HDT to treat persistent infection

**Figure 3:**
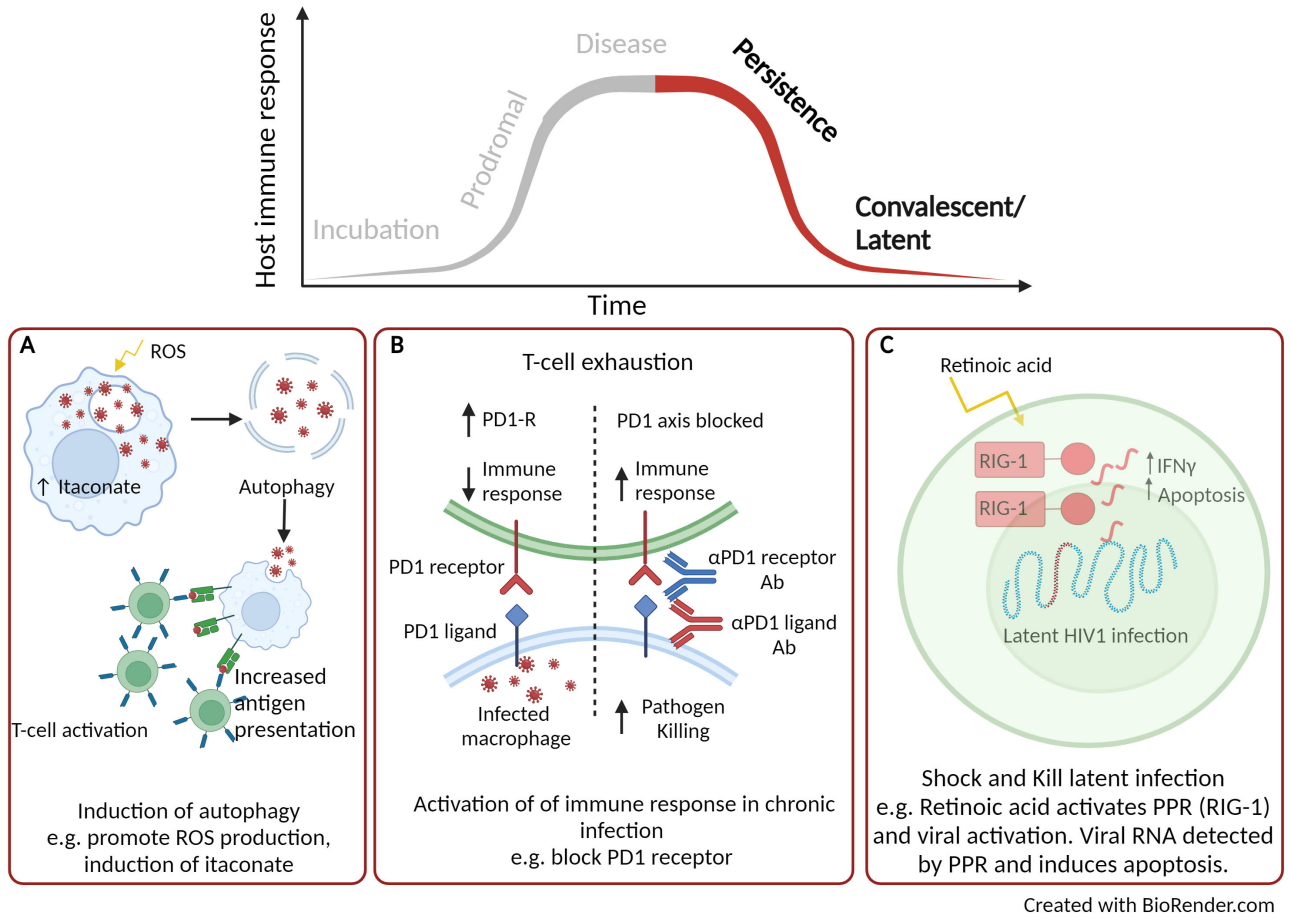
Using host directed therapy to treat persistence and latent infection. **(A)** Activation of autophagy. **(B)** Inhibition of immune checkpoints. **(C)** Shock and kill therapy. Created with BioRender.com.

Persistent infections are described as those in which the pathogen is not cleared during the primary infection and can remain viable within the host. There are three overlapping types of persistence, defined as chronic, slow and latent infection. Here we described the potential use of HDTs to target the various stages of persistency. *M. tuberculosis*, is well adapted to persist infection and resides in phagosomes of the infected macrophage. Here the pathogen inhibits phagosomal fusion and slowly replicates, evading the host response, leading to chronic infection and tissue pathology if left untreated (128, 129). HDTs have been identified to activate autophagy and is an area of interest for the treatment of intracellular bacterial pathogens including mycobacteria (**Figure 3A**) (130). A number of small compounds can be used to induce autophagy, for example activation of ROS, blocking ion channels and maturation of the phagosome. Autophagy allows the release of infectious particles, which can then be taken up by activated phagocytic cells (131). Rapamycin is a broad range anti-inflammatory drug originally approved for the use of organ transplant rejection (132). Rapamycin has been extensively studied as an inducer of autophagy (133) and *in vivo M. tuberculosis* infection models have demonstrated reduced mycobacterial lung immunopathology, the formation of necrotic lesions within the lung (134) and clearance of mycobacteria, including multi-drug resistant strains (135). Similar autophagy inducing drugs, such as ridaforolimus (approved for use in the treatment of solid tumours and haematological malignancies (136)) and temsirolimus [approved for use in renal cell carcinoma therapy (137)] have demonstrated potential therapeutic benefits for the treatment of tuberculosis (138). Furthermore, the repurposing of metformin has also been shown to support macrophage control through the induction of ROS and has been shown to improve the resolution of lung cavities in patients with tuberculosis (139). Itaconate, as described earlier is a broad-ranging anti-inflammatory host molecule that has also been shown to regulate autophagy through activation of the transcription factor EB (TFEB). Antimicrobial activity of the induced endogenous metabolite has been reported to limit infection of intracellular bacteria *Salmonella typhimurium* infection *in vitro* and *in vivo* (140, 141).

Immune checkpoints are signalling pathways that regulate the host immune response. They are critical for self-tolerance but are also activated during chronic inflammatory responses, such as sepsis and during persistent infection. Once activated, the immune response is dampened which can alleviate immune-directed tissue damage but can also reduce the effectiveness of clearing the infection (142). There are a variety of interactions between antigen presenting cells and T-cells that can promote T-cell exhaustion leading to inhibitory effects of the immune response and these are illustrated in a previous review (142). Once such interaction is that of the co-inhibitory receptor, programmed death-1 (PD1) expressed on T-cells and its corresponding ligand (PDL-1) found on dendritic cells. A number of approved inhibitors targeting these checkpoint proteins are in use for cancer immunotherapy such as PD-1 inhibitors (Nivolumab, Pembrolizumab and Cemiplimab), PDL-1 inhibitors (Atezolizumab, Durvalumab and Avelumab) (143) and are now being considered for the treatment of viral infections (**Figure 3B**). In simian immunodeficiency viruses (SIV)-infected macaques, treatment with a humanised anti-PD1 antibody led to improved functionality of CD8^+^ T cells, reduced amounts of SIV RNA and increased survival of the macaques (144). Further beneficial efficacy has been described using anti-PD1 or anti-PDL-1 for the treatment of hepatitis B and C in pre-clinical infection models (145, 146). When blocking the PD1/PD1-L interaction, IFNγ production was no longer suppressed, anti-viral T-cell phenotypes were restored and there was significant clearance of viral persistence (145, 146). While blocking immune checkpoints for viral infection has shown beneficial therapeutic effects, these effects can be detrimental in chronic bacterial disease, such as tuberculosis. Using PD1 deficient murine model infected with *M. tuberculosis* led to significantly reduced survival (147, 148), uncontrolled bacterial proliferation with areas necrotic foci (149), compared to infection of wild-type mice. Further, there was increased number of neutrophils and high levels of TNFα and IL-6 which corresponded to a discordant inflammatory response (149). These studies highlight that such HDT is not necessarily appropriate for intracellular bacterial infection and that consideration and understanding of immunopathology is a critical consideration.

Latent infection is another area of research where HDT could be utilised to treat disease, in particular this has been described for HIV-1. The approach used is termed “Shock and Kill”, where latency reversal agents actively induce replication of latent HIV-1 and thus making the infectious viral particles more susceptible to clearance through the host immune response (**Figure 3C**) (150). Retinoids (a derivative of Vitamin A) have been long approved for the treatment of a number of cancers as well as various skin conditions (151) and are now been considered for latent HIV-1 therapy. Retinoids have been shown to re-activate virus replication by activating the PRR, RIG-1 (152), which detects viral RNA (153). Once viral RNA is detected by PRRs, CD8^+^ cytotoxic T-cells are induced which have enhanced anti-viral properties and can eliminate infected cells (154). Although there is concern that the “Shock and Kill” approach may increase permissiveness of HIV-1 infection, used in combination with standard HIV-1 therapy may make this a beneficial therapeutic approach (155). The unique use of retinoids as latency reversal agents which can activate viral replication alongside anti-viral activity could also have the potential to treat a range of quiescence viral infections.

## 5 Summary of the use of HDT for infectious disease and future direction

**Figure 4:**
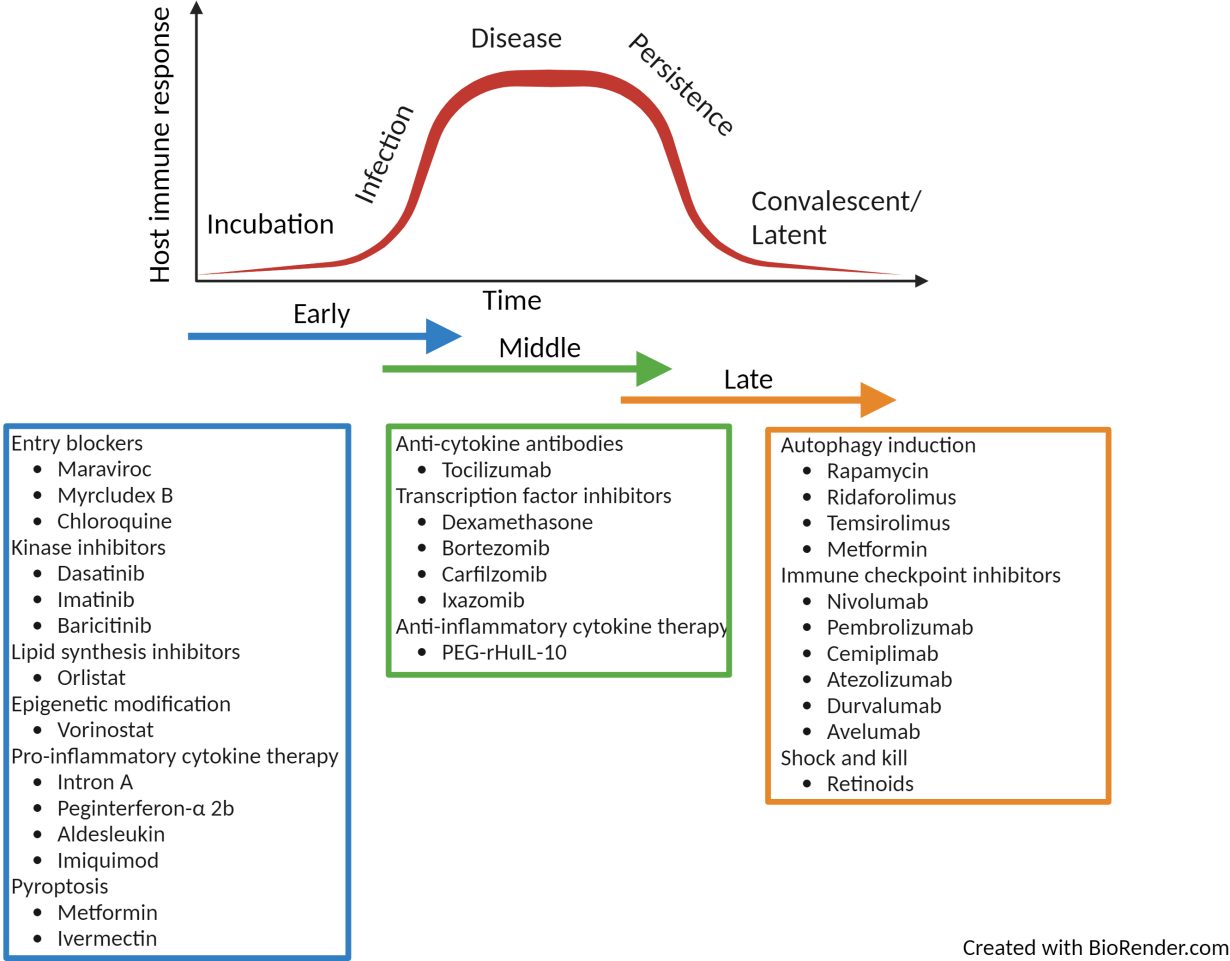
Summary of licenced drugs approved for clinical use in the treatment of immune-related diseases. The summary of drugs listed is not comprehensive but highlights a range of therapies that could be repurposed as HDT for infectious disease over the time-course of infection. Created with BioRender.com.

HDTs represent a novel solution for the treatment of infectious disease. Their immunomodulatory action make them ideal for combatting the spread of antimicrobial resistance as well as emerging new pathogens. Cancer HDTs are leading the way; where in 2021 there were 14 immunomodulators, 20 cellular and gene therapies and 98 antibody therapies currently approved by the United States Food and Drug Administration (156). These compounds have huge potential to be repurposed for the treatment of infectious disease. Currently much of the focus has been on discovering HDTs for tuberculosis, hepatitis B and C and HIV-1 but due to their pleiotropic functions, HDTs have huge promise for the treatment of a broad range of infectious disease. Throughout the review, examples of clinically approved licenced drugs for the treatment of immune related diseases have been described and these have been summarised in **Figure 4**. The summary is not exhaustive but lists a number or approved therapies that could be repurposed. The repurposing of such drugs have huge potential as they already have well established safety and pharmokinetic profiles as well as known manufacturing and distribution networks. The use of such therapies mean they could become quickly available for alternative indications. Although HDTs have many advantages over pathogen-directed anti-microbial treatment, (for example, reduced likelihood of the development of resistant microbial strains and potential broad-spectrum use), it is more likely that these therapies would be used as part of a layered defence strategy in combination with other anti-microbial therapies.

In this review, we described potential targets for HDT over the trajectory of infection, from entry of pathogen, through disease and followed by persistence and latency. Over the time course of disease there are a range of immune related pathways that could be targeted, and typically a HDT is targeted to a specific phase of infection. The downstream effect of the therapy is dependent on whether a pathway is being blocked or activated. An agonist or an antagonist to particular receptors can completely alter the response and therefore outcome of disease. To avoid inadvertently manipulating an immune response pathway that would be detrimental to the host, it is highly likely that immune-profiling diagnostics would be required to help identify and characterise the patient’s stage of disease. Indeed, these time dependent HDT approaches are limited as they do not allow flexibility to manage disease through the course of an infection.

In more recent years, the understanding of immune function pathways are now becoming well characterised and offer some of the most exciting opportunities for HDT development. Alongside the use of companion diagnostics, emerging therapies have been identified that could either augment or dampen a specific pathway depending on the stage of infection and inflammatory response. For instance, activation of down-stream PAMP signalling such as that described for cGAS-STING could support an early innate host response (**Figure 1D**), but later in the disease profile, antagonists of this pathway may reduce immune-pathological tissue damage (**Figure 2B**). Similarly, HDTs that can induce inflammasomes, pro-inflammatory cytokine release and rapid programmed cell death (e.g. pyroptosis) are beneficial in the early stages of infection (**Figure 1D**), but as disease progresses it would be more beneficial to inhibit such pathways (**Figure 2A**). Furthermore, the increased understanding of the intricate link between cellular metabolism and immune function reveals potential pathways that could be targeted by HDT. For example, inhibition of the TCA metabolite, itaconate, prevents both the utilization as a carbon source to support bacterial growth as well as the induction of an early anti-inflammatory immune response (**Figure 1B**). However, the immune-modulatory properties of itaconate can be of benefit later in disease where enhancing this pathway would support the host response (**Figures 2A–C**, **3A**). Research of such immune functions in healthy and disease state are still in their infancy and it is critical to understand the pharmacokinetics of such compounds that can enhance or reduce such pathways. The ability to refine and modify an immune-regulated pathway to manage infection across the disease profile would be incredibly beneficial.

Looking forward, in a generation of systems biology and the huge advances in “omics” technology (for example, transcriptomics, epigenetics, metabolomics and proteomics), high-throughput immune profiling has the potential to identify an individual’s susceptibility to infection (157) and long term-prognosis (158). The use of patient specific “omic” data alongside microbial whole-genome sequencing and machine learning would be indispensable for the future of evidence-based management of infectious disease and precision medicine. The bespoke application of HDT to modulate a patient’s immune response in combination with antimicrobial drug therapy is the future to treating infectious disease and the management of drug-resistant pathogens.
